# Supplementary material for: Pregnancy Outcomes in Women with Biventricular Circulation and a Systemic Right Ventricle: A Systematic Review
Source: J Clin Med. 2024 Nov 29;13(23):7281. doi: 10.3390/jcm13237281 (PMC11642114; doi:10.3390/jcm13237281)

Supplementary Materials

Pregnancy Outcomes in Women with Biventricular Circulation and a Systemic Right Ventricle: A Systematic Review

Triantafyllia Grantza [1], Alexandra Arvanitaki [1,2], Amalia Baroutidou [1], Ioannis Tsakiridis [3], Apostolos Mamopoulos [3], Andreas Giannopoulos [4], Antonios Ziakas [1], George Giannakoulas [1]

[1] First Department of Cardiology, AHEPA University General Hospital, School of Medicine, Aristotle University of Thessaloniki, 54636 Thessaloniki, Greece

[2] Adult Congenital Heart Centre and National Centre for Pulmonary Hypertension, Royal Brompton and Harefield Hospitals, Guy's and St Thomas's NHS Foundation Trust, Imperial College, London SW3 5NP, United Kingdom.

[3] 3rd Department of Obstetrics and Gynecology, Hippokratio General Hospital, School of Medicine, Aristotle University of Thessaloniki, 54642 Thessaloniki, Greece.

[4] Pediatric Department, Aristotle University of Thessaloniki, AHEPA University General Hospital, School of Medicine, 54636 Thessaloniki, Greece.

Contents

Section S1. Search Strategy 3

PubMed Syntax 3

CENTRAL Syntax 4

Table S1. Reasons for excluding records at full-text level 5

Figure S1. PRISMA 2020 flow diagram for new systematic reviews which included searches of databases and registers only 8

Table S2. Quality assessment of the present systematic review 9

Table S3. Risk of bias assessment of the included studies according to the Newcastle-Ottawa Scale. 12

Figure S2. Publication bias. 13

Figure S3. Subgroup analysis for outcomes without statistically significant difference between d-TGA and ccTGA group. 15

Section S1. Search Strategy

PubMed Syntax

Search terms for population:

#1 Transposition of the Great Arteries

#2 Transposition of the Great Vessels

#3 TGA

#4 dTGA

#5 Atrial switch procedure

#6 Mustard procedure

#7 Senning procedure

#8 Congenitally corrected transposition of the Great Arteries

#9 ccTGA

#10 L-TGA

#11 Systemic Right Ventricle

#12 Systematic right ventricle

#13 [(#1 OR #2 OR #3 OR #4) AND (#5 OR #6 OR #7) OR (#8 OR #9 OR #10)] OR (#11 OR #12)

b. Search terms for intervention

#14 pregnancy

#15 pregnant

#16 (#14 OR #15)

c.

#17 (#13 AND #16)

Search:

(((((((transposition of the great arteries) OR (transposition of the great vessels)) OR (TGA)) OR (dTGA)) AND (((Atrial switch procedure) OR (Mustard procedure)) OR (Senning procedure))) OR (((congenitally corrected transposition of the great arteries) OR (ccTGA)) OR (L-TGA))) OR ((Systemic Right Ventricle) OR (Systematic Right Ventricle))) AND ((pregnancy[MeSH Terms]) OR (pregnant woman))

((("transposition of great vessels"[MeSH Terms] OR ("transposition"[All Fields] AND "great"[All Fields] AND "vessels"[All Fields]) OR "transposition of great vessels"[All Fields] OR ("transposition"[All Fields] AND "great"[All Fields] AND "arteries"[All Fields]) OR "transposition of the great arteries"[All Fields] OR ("transposition of great vessels"[MeSH Terms] OR ("transposition"[All Fields] AND "great"[All Fields] AND "vessels"[All Fields]) OR "transposition of great vessels"[All Fields] OR "transposition of the great vessels"[All Fields]) OR "TGA"[All Fields] OR "dTGA"[All Fields]) AND ("arterial switch operation"[MeSH Terms] OR ("arterial"[All Fields] AND "switch"[All Fields] AND "operation"[All Fields]) OR "arterial switch operation"[All Fields] OR ("atrial"[All Fields] AND "switch"[All Fields] AND "procedure"[All Fields]) OR "atrial switch procedure"[All Fields] OR ("arterial switch operation"[MeSH Terms] OR ("arterial"[All Fields] AND "switch"[All Fields] AND "operation"[All Fields]) OR "arterial

switch operation"[All Fields] OR ("mustard"[All Fields] AND "procedure"[All Fields]) OR "mustard procedure"[All Fields]) OR ("arterial switch operation"[MeSH Terms] OR ("arterial"[All Fields] AND "switch"[All Fields] AND "operation"[All Fields]) OR "arterial switch operation"[All Fields] OR ("senning"[All Fields] AND "procedure"[All Fields]) OR "senning procedure"[All Fields]))) OR ("congenitally corrected transposition of the great arteries"[MeSH Terms] OR ("congenitally"[All Fields] AND "corrected"[All Fields] AND "transposition"[All Fields] AND "great"[All Fields] AND "arteries"[All Fields]) OR "congenitally corrected transposition of the great arteries"[All Fields] OR "ccTGA"[All Fields] OR "L-TGA"[All Fields]) OR ((("systemic"[All Fields] OR "systemically"[All Fields] OR "systemics"[All Fields]) AND ("heart ventricles"[MeSH Terms] OR ("heart"[All Fields] AND "ventricles"[All Fields]) OR "heart ventricles"[All Fields] OR ("right"[All Fields] AND "ventricle"[All Fields]) OR "right ventricle"[All Fields])) OR (("classification"[MeSH Terms] OR "classification"[All Fields] OR "systematic"[All Fields] OR "classification"[MeSH Subheading] OR "systematics"[All Fields] OR "systematical"[All Fields] OR "systematically"[All Fields] OR "systematisation"[All Fields] OR "systematise"[All Fields] OR "systematised"[All Fields] OR "systematization"[All Fields] OR "systematizations"[All Fields] OR "systematize"[All Fields] OR "systematized"[All Fields] OR "systematizes"[All Fields] OR "systematizing"[All Fields]) AND ("heart ventricles"[MeSH Terms] OR ("heart"[All Fields] AND "ventricles"[All Fields]) OR "heart ventricles"[All Fields] OR ("right"[All Fields] AND "ventricle"[All Fields]) OR "right ventricle"[All Fields])))) AND ("pregnancy"[MeSH Terms] OR ("pregnant women"[MeSH Terms] OR ("pregnant"[All Fields] AND "women"[All Fields]) OR "pregnant women"[All Fields] OR ("pregnant"[All Fields] AND "woman"[All Fields]) OR "pregnant woman"[All Fields]))

CENTRAL Syntax

#1	Transposition of the Great arteries
#2	transposition of the great vessels
#3	d-TGA
#4	atrial switch
#5	mustard procedure
#6	senning procedure
#7	congenitally corrected transposition of the great arteries
#8	ccTGA
#9	systemic right ventricle
#10	systematic right ventricle
#11	pregnancy
#12	pregnant
#13	(#1 OR #2 OR #3)
#14	(#4 OR #5 OR #6)
#15	(#7 OR #8)
#16	(#9 OR #10)
#17	(#11 OR #12)
#18	(#13 AND #14)
#19	(#18 OR #15 OR #16)

#20 (#19 AND #17)

Table S1. Reasons for excluding records at full-text level

	Study	Reason for excluding
1.	S Mital; Right ventricle in congenital heart disease: is it just a "weaker" left ventricle?	Wrong study design
2.	E Kowalik; Management of congenitally corrected transposition from fetal diagnosis to adulthood.	Not reporting the outcome of interest
3.	Raffaella Marzullo, Magalie Ladouceur, Gianpiero Gaio, Mario Giordano, Maria Giovanna Russo, Berardo Sarubbi; Impact of pregnancy on natural history of systemic right ventricle in women with transposition of the great arteries.	Wrong study design
4.	Tim S Hornung, Louise Calder; Congenitally corrected transposition of the great arteries.	Unavailable full-text
5.	R D'Souza, C Silversides; Pregnancy following atrial-switch repair.	Wrong study design
6.	Daniel J Murphy Jr; Transposition of the great arteries: long-term outcome and current management. Curr Cardiol Rep. 2005 Jul;7(4):299-304	Not reporting the population of interest
7.	Daniel Tobler 1, Susan M Fernandes, Rachel M Wald, Michael Landzberg, Omid Salehian, Samuel C Siu, Jack M Colman, Matthew Sermer, Candice K Silversides; Pregnancy outcomes in women with transposition of the great arteries and arterial switch operation. Am J Cardiol. 2010 Aug 1;106(3):417-20.	Not reporting the population of interest
8.	J Soongswang, I Adatia, C Newman, J F Smallhorn, W G Williams, R M Freedom; Mortality in potential arterial switch candidates with transposition of the great arteries. J Am Coll Cardiol. 1998 Sep;32(3):753-7.	Not reporting the outcome of interest
9.	G Megerian, J G Bell, J C Huhta, J N Bottalico, S Weiner; Pregnancy outcome following Mustard procedure for transposition of the great arteries: a report of five cases and review of the literature. 1994 Apr;83(4):512-6.	Wrong study design
10.	Torri D Metz, G Marc Jackson, Anji T Yetman; Pregnancy outcomes in women who have undergone an atrial switch repair for congenital d-transposition of the great arteries. Am J Obstet Gynecol. 2011 Sep;205(3):273.e1-5.	Incomplete data
11.	Emily E Naoum, Jamel P Ortoleva, Ryan M Militana, Marti D Soffer, Doreen DeFaria Yeh; Anesthesia for cesarean delivery in a patient with congenitally corrected transposition of the great arteries: A case report. Ann Card Anaesth. 2023 Oct-Dec;26(4):446-450.	Wrong study design

12.	K E Woodson, C A Sable, J T Berger 3rd, M C Slack, G Wernovsky, T L Spray; A case of congenitally protected d-transposition of the great arteries in a very low-birth-weight infant. Pediatr Cardiol. 2003 Mar-Apr;24(2):175-8.	Not reporting the population of interest
13.	M P Rousseil, O Irion, F Béguin, O Jaques, R Adamec, R Lerch, B Friedli, K Rifat; Successful term pregnancy after Mustard operation for transposition of the great arteries. Eur J Obstet Gynecol Reprod Biol. 1995 Mar;59(1):111-3.	Wrong study design
14.	R Jalalian, S Masoumi, A Ghaemian; Diagnosis of a congenitally corrected transposition of the great arteries in a 50-year-old multiparous woman. Cardiovasc J Afr. 2011 Jul-Aug;22(4):203-4.	Wrong study design
15.	Muhammad Kamran Younis Memon, Ayesha Malik, Saleem Akhtar; Successful completion of pregnancy after Mustard procedure for transposition of great arteries: a rare case from the developing world. J Pak Med Assoc. 2020 May;70(5):920-922.	Wrong study design
16.	E H Dellinger, H A Hadi; Maternal transposition of the great arteries in pregnancy. A case report. J Reprod Med. 1994 Apr;39(4):324-6.	Wrong study design
17.<br>18.	H Reinecke, U Cirkel, S Kerber, S Kotthoff, F Louwen, T Wichter, G Breithardt; Pregnancy in patients with transposition of great vessels corrected by the Mustard procedure. Report of a case and review of reported cases. Z Kardiol<br>. 1997 Nov;86(11):945-56.	Foreign Language
19.	Victoria M Stoll, Nigel E Drury, Sara Thorne, Tara Selman, Paul Clift, Hsu Chong, Peter J Thompson, R Katie Morris, Lucy E Hudsmith; Pregnancy Outcomes in Women with Transposition of the Great Arteries After an Arterial Switch Operation. JAMA Cardiol. 2018 Nov 1;3(11):1119-1122.	Not reporting the population of interest
20.	Hacer Ceren Tokgöz, Özgür Yaşar Akbal, Ali Karagöz, Barkın Kültürsay, Seda Tanyeri, Berhan Keskin, Aykun Hakgör, Şeyhmus Külahçıoğlu, Zübeyde Bayram, Süleyman Çağan Efe, Cem Doğan, İbrahim Halil Tanboğa, Nihal Özdemir, and Cihangir Kaymaz; Maternal and Fetal Outcomes in Pregnant Women with Pulmonary Arterial Hypertension: A Single-Center Experience and Review of Current Literature. Anatol J Cardiol. 2022 Dec; 26(12): 902–913.	Not reporting the population of interest
21.	Lindley, K.J., Bairey Merz, C.N., Asgar, A.W., Bello, N.A., Chandra, S., Davis, M.B., Gomberg-Maitland, M., Gulati, M., Hollier, L.M., Krieger, E.V., Park, K., Silversides, C., Wolfe, N.K., Pepine, C.J.; Management of Women With Congenital or Inherited Cardiovascular Disease From Pre-Conception	Wrong study design

	Through Pregnancy and Postpartum: JACC Focus Seminar 2/5. J Am Coll Cardiol. 2021 Apr 13;77(14):1778-1798.	
22.	Sarah A. Goldstein, MD, Lisa Sorenson, CNP, Jeffrey B. Chapa, MD and Richard A. Krasuski, MD; Pregnancy in a woman with congenitally corrected transposition of the great arteries. Cleveland Clinic Journal of Medicine April 2021, 88 (4) 228-236.	Wrong study design
23.	Louise F Udholm, Ninna H Ebdrup, Linn H Arendt, Ulla B Knudsen, Vibeke E Hjortdal, Cecilia H Ramlau-Hansen; Congenital heart disease and the risk of impaired fertility: A Danish nationwide cohort study using time to pregnancy. Int J Cardiol. 2023 Aug 1:384:25-30.	Not reporting the population of interest
24.	Curt J Daniels; The development of adult congenital heart disease care in the United States. Curr Probl Pediatr Adolesc Health Care. 2023 Mar;53(3):101400.	Not reporting the outcome of interest
25.	Virginija Rudienė, Lina Kaplerienė, Dovilė Jančauskaitė, Emilija Meškėnė, Eglė Palevičiūtė, Monika Laukytė-Slėnienė, Diana Gasiūnaitė, Diana Ramašauskaitė, Elena Jurevičienė, and Lina Gumbienė; Pregnancy in Congenital Heart Disease, Complicated by Pulmonary Arterial Hypertension—A Challenging Issue for the Pregnant Woman, the Foetus, and Healthcare Professionals. Medicina (Kaunas). 2022 Apr; 58(4): 476.	Not reporting the population of interest
26.	Candice K. Silversides, Jack M. Colman, Samuel C. Siu; Congenital Heart Disease and Pregnancy. J Am Coll Cardiol. 2018 May 29;71(21):2419-2430.	Incomplete data
27.	Avila, W.S., Ribeiro, V.M., Rossi, E.G., Binotto, M.A., Bortolotto, M.R., Testa, C., Francisco, R., Hajjar, L.A., Miura, N.; Pregnancy in women with complex congenital heart disease. A constant challenge. Arq Bras Cardiol. 2019 Dec;113(6):1062-1069.	Not reporting the population of interest
28.	Koichiro Niwa; Adult Congenital Heart Disease with Pregnancy. Korean Circ J. 2018 Apr;48(4):251-276.	Wrong study design
29.	Cuenca Marín, C., Ibrahim Díez, N., Armenteros Pérez, A., González Mesa, E.; Pregnancy in a patient with d-transposition of large arteries undergoing surgical correction. Progresos de Obstetricia y Ginecologia, September-October 2017, Pages 458-461	Unavailable full text
30.	Matthew Cauldwell, Michael Gatzoulis, and Philip Steer; Congenital heart disease and pregnancy: A contemporary approach to counselling, pre-pregnancy investigations and the impact of pregnancy on heart function. Obstet Med. 2017 Jun; 10(2): 53–57.	Not reporting the outcome of interest
31.	Ami B Bhatt, Doreen DeFaria Yeh; Pregnancy and Adult Congenital Heart Disease. Cardiol Clin. 2015 Nov;33(4):611-23.	Not reporting the population of interest

32.	Olga H Balint 1, Samuel C Siu, Jennifer Mason, Jasmine Grewal, Rachel Wald, Erwin N Oechslin, Brigitte Kovacs, Mathew Sermer, Jack M Colman, Candice K Silversides; Cardiac outcomes after pregnancy in women with congenital heart disease. Heart. 2010 Oct;96(20):1656-61.	Not reporting the population of interest
33.	Marie Pank, Signe Holm Larsen, Keld Sørensen, Vibeke Hjortdal; Pregnancy after Mustard operation for transposition of the great arteries. Ugeskr Laeger. 2009 Feb 16;171(8):602-6.	Foreign Language

Figure S1. PRISMA 2020 flow diagram for new systematic reviews which included searches of databases and registers only

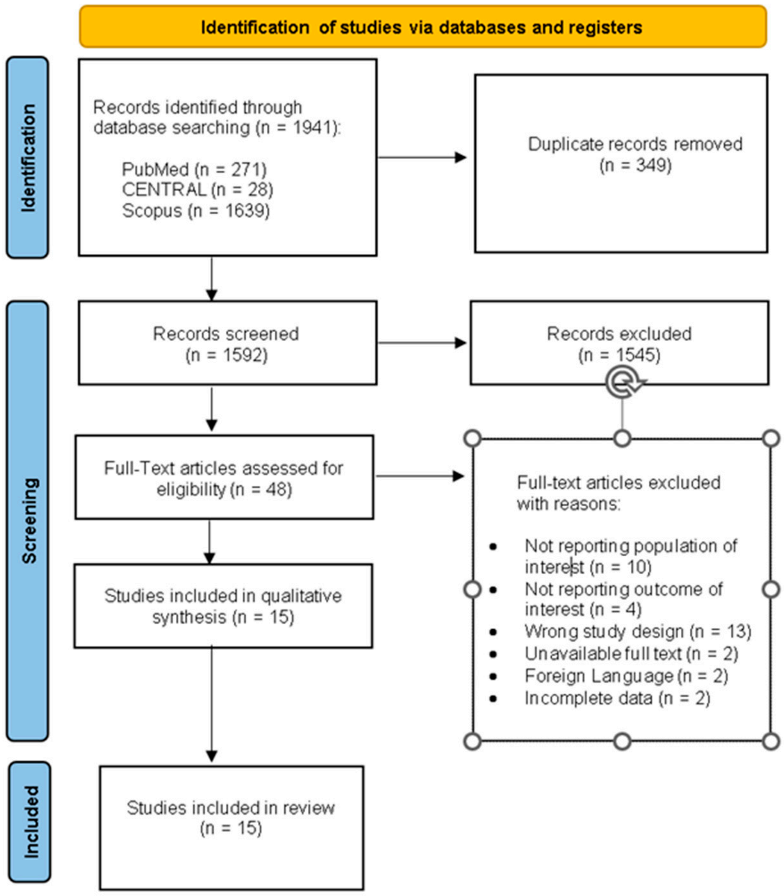

Table S2. Quality assessment of the present systematic review [6].

Section and	Item	Checklist item	Location where item is reported
**TITLE**			
Title	1	Identify the report as a systematic review.	Page 1
**ABSTRACT**			
Abstract	2	See the PRISMA 2020 for Abstracts checklist.	Page 2
**INTRODUCTION**			
Rationale	3	Describe the rationale for the review in the context of existing knowledge.	Page 2
Objectives	4	Provide an explicit statement of the objective(s) or question(s) the review addresses.	Page 2
**METHODS**			
Eligibility criteria	5	Specify the inclusion and exclusion criteria for the review and how studies were grouped for the syntheses.	Page 3
Informatio n sources	6	Specify all databases, registers, websites, organizations, reference lists and other sources searched or consulted to identify studies. Specify the date when each source was last searched or consulted.	Page 2, Appendix S1
Search strategy	7	Present the full search strategies for all databases, registers and websites, including any filters and limits used.	Page 2, Appendix S1
Selection process	8	Specify the methods used to decide whether a study met the inclusion criteria of the review, including how many reviewers screened each record and each report retrieved, whether they worked independently, and if applicable, details of automation tools used in the process.	Page 3
Data collection process	9	Specify the methods used to collect data from reports, including how many reviewers collected data from each report, whether they worked independently, any processes for obtaining or confirming data from study investigators, and if applicable, details of automation tools used in the process.	Page 3
Data items	10a	List and define all outcomes for which data were sought. Specify whether all results that were compatible with each outcome domain in each study were sought (e.g. for all measures, time points, analyses), and if not, the methods used to decide which results to collect.	Page 3
	10b	List and define all other variables for which data were sought (e.g. participant and intervention characteristics, funding sources). Describe any assumptions made about any missing or unclear information.	Pages 3-5
Study risk of bias	11	Specify the methods used to assess risk of bias in the included studies, including details of the tool(s) used, how many reviewers assessed each study and whether they worked independently, and if applicable, details of automation tools used in the process.	Page 3, Figure S2
Effect measures	12	Specify for each outcome the effect measure(s) (e.g. risk ratio, mean difference) used in the synthesis or presentation of results.	Page 4

Synthesi s methods	13a	Describe the processes used to decide which studies were eligible for each synthesis (e.g. tabulating the study intervention characteristics and comparing against the planned groups for each synthesis (item #5)).	Page 3
	13b	Describe any methods required to prepare the data for presentation or synthesis, such as handling of missing summary statistics, or data conversions.	Page 3
	13c	Describe any methods used to tabulate or visually display results of individual studies and syntheses.	Page 3
	13d	Describe any methods used to synthesize results and provide a rationale for the choice(s). If meta-analysis was performed, describe the model(s), method(s) to identify the presence and extent of statistical heterogeneity, and software package(s) used.	Page 3
	13e	Describe any methods used to explore possible causes of heterogeneity among study results (e.g. subgroup analysis, meta-regression).	Page 4
	13f	Describe any sensitivity analyses conducted to assess robustness of the synthesized results.	Page 4
Reporting bias assessment	14	Describe any methods used to assess risk of bias due to missing results in a synthesis (arising from reporting biases).	Page 3, Figure S2
Certainty assessme	15	Describe any methods used to assess certainty (or confidence) in the body of evidence for an outcome.	Page 3
**RESULTS**			
Study selection	16a	Describe the results of the search and selection process, from the number of records identified in the search to the number of studies included in the review, ideally using a flow diagram.	Pages 4-14
	16b	Cite studies that might appear to meet the inclusion criteria, but which were excluded, and explain why they were excluded.	Page 14, Table S1
Study characteristics	17	Cite each included study and present its characteristics.	Pages 4-6
Risk of bias in studies	18	Present assessments of risk of bias for each included study.	Page 14, Figure S2
Results of individual studies	19	For all outcomes, present, for each study: (a) summary statistics for each group (where appropriate) and (b) an effect estimate and its precision (e.g. confidence/credible interval), ideally using structured tables or plots.	Pages 8-10, 12-13
Results of syntheses	20a	For each synthesis, briefly summarise the characteristics and risk of bias among contributing studies.	Pages 6-7, 11
	20b	Present results of all statistical syntheses conducted. If meta-analysis was done, present for each the summary estimate and its precision (e.g. confidence/credible interval) and measures of statistical heterogeneity. If comparing groups, describe the direction of the effect.	Pages 4-14
	20c	Present results of all investigations of possible causes of heterogeneity among study results.	Pages 7-8, 11-12, 14

	20d	Present results of all sensitivity analyses conducted to assess the robustness of the synthesized results.	Pages 8, 12, 14
Reporting biases	21	Present assessments of risk of bias due to missing results (arising from reporting biases) for each synthesis assessed.	Pages 14, 16, Figure S2
Certainty of evidence	22	Present assessments of certainty (or confidence) in the body of evidence for each outcome assessed.	Page 7-8, 11-12, 14
**DISCUSSION**			
Discussion	23a	Provide a general interpretation of the results in the context of other evidence.	Page 14
	23b	Discuss any limitations of the evidence included in the review.	Page 16
	23c	Discuss any limitations of the review processes used.	Page 16
	23d	Discuss implications of the results for practice, policy, and future research.	Page 16
**OTHER INFORMATION**			
Registration and protocol	24a	Provide registration information for the review, including register name and registration number, or state that the review was not registered.	Page 2
	24b	Indicate where the review protocol can be accessed, or state that a protocol was not prepared.	Page 2
	24c	Describe and explain any amendments to information provided at registration or in the protocol.	Page 2
Support	25	Describe sources of financial or non-financial support for the review, and the role of the funders or sponsors in the review.	Page 17
Competing interests	26	Declare any competing interests of review authors.	Page 17
Availability of data, code and other materials	27	Report which of the following are publicly available and where they can be found: template data collection forms; data extracted from included studies; data used for all analyses; analytic code; any other materials used in the review.	Page 17

Table S3. Risk of bias assessment of the included studies according to the Newcastle- Ottawa Scale.

	Selection Overall				Comparability	Outcome Overall			Overall Score
**Author, Year**	**Representativeness**	**Selection of non-exposed**	**Exposure ascertainment**	**Outcome absent at baseline**		**Outcome assessment**	**Length of follow-up**	**Adequacy of follow-up**	
**Connolly, 1999**	★	NA	★	NA	NA	★	★	★	5
**Therrien, 1999**	★	NA	★	NA	★	★	★	★	6
**Guedes, 2004**	★	NA	★	NA	NA	★	★	★	5
**Canobbio, 2006**	★	NA	★	NA	NA	★	★	★	5
**Gelson, 2011**	★	NA	★	NA	★	★	★	★	6
**Jain, 2011**	★	NA	★	NA	★	★	★	★	6
**Tutarel, 2011**	★	NA	★	NA	★	★	★	★	5
**Zentner, 2012**	★	NA	★	NA	★	★	★	★	5
**Bowater, 2013**	★	NA	★	NA	NA	★	★	★	5
**Kowalik, 2014**	★	NA	★	NA	NA	★	★	★	5
**Cataldo, 2015**	★	NA	★	NA	★	★	★	★	6
**Lipczyńska, 2016**	★	NA	★	NA	NA	★	★	★	5
**Frabre-Gray, 2019**	★	NA	★	NA	★	★	★	★	6
**Yang, 2020**	★	NA	★	NA	NA	★	★	★	6
**Harada, 2022**	★	NA	★	NA	NA	★	★	★	5

NA (Not applicable)

The Newcastle-Ottawa scale uses a nine-star rating system to assess the study quality with regard to 3 domains: Selection of the study groups (including 4 items), Comparability of the groups (including 1 item), and Ascertainment of the outcome of interest (including 3 items). Each item within the Selection and

Outcome domains can be rated with maximum one star, whereas Comparability can be rated with maximum two stars. Overall NOS scores range from 0 to 9, with 9 indicating the highest possible quality. Overall scores 7-9, 4-6, and <4 were regarded as low, intermediate and high risk, respectively.

Figure S2. Publication bias.

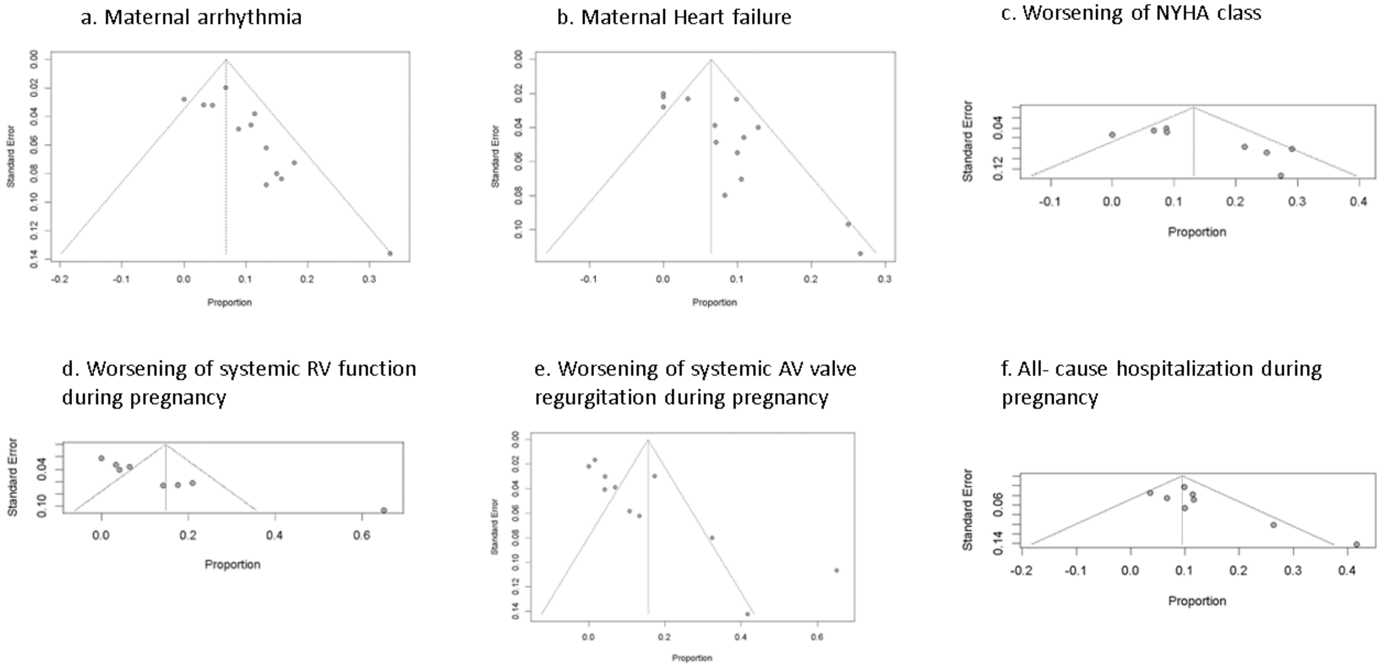

NYHA class: New York Heart Association classification; RV: right Ventricle; AV valve: atrioventricular valve

a. Maternal Arrhythmia. The visual inspection of the funnel plot for arrhythmia during pregnancy displayed asymmetry of studies. Egger's regression test also confirmed the presence of potential publication bias (t= 3.61, p-value= 0.0041).

b. Maternal Heart Failure. The visual inspection of the funnel plot for maternal heart failure during pregnancy displayed asymmetry of studies. The Egger's regression test demonstrated significant evidence of publication bias (t= 3.40, p-value= 0.053).

c. Worsening of NYHA class. The funnel plot for the Worsening of NYHA class during pregnancy did not reveal severe asymmetry. However, the Egger's regression test demonstrated significant evidence of publication bias (t= 3.13, p-value= 0.02).

d. Worsening of systemic RV function during pregnancy. The visual inspection of the funnel plot for worsening of systemic RV function during pregnancy displayed asymmetry of studies. Egger's regression test also confirmed the presence of potential publication bias (t= 5.79, p-value= 0.0012).

e. Worsening of systemic AV valve regurgitation during pregnancy. The visual inspection of the funnel plot for worsening of systemic RV function during pregnancy displayed asymmetry of studies. The Egger's regression test demonstrated significant evidence of publication bias (t= 3.85, p-value= 0.0039).

f. All cause hospitalization during pregnancy. The funnel plot for the Worsening of NYHA class during pregnancy did not reveal severe asymmetry. Also, Egger's regression test confirmed no significant evidence of publication bias (t= 2.07, p-value = 0.0842).

Figure S3. Subgroup analysis for outcomes without statistically significant difference between d-TGA and ccTGA group.
d-TGA: dextro-looped Transposition of the Great Arteries; ccTGA: Congenitally Corrected Transposition of the Great Arteries; RV: Right Ventricle; AV valve: Atrioventricular Valve; SGA: Small for Gestational Age. [15,16,18–20,22–26,31,43].

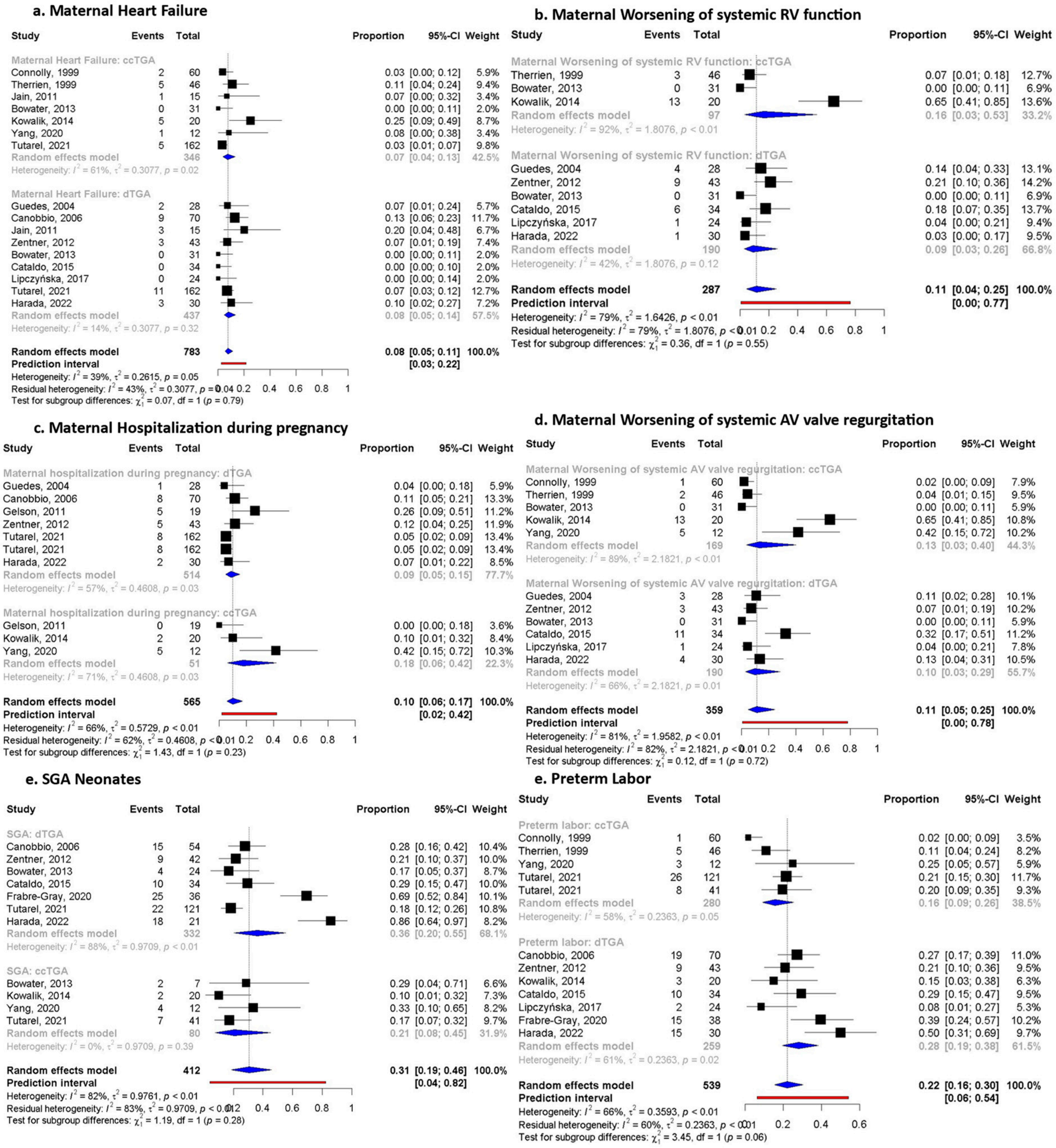

Supplement: Supplementary file 1 [file jcm-13-07281-s001.zip › jcm-3311134-supplementary.pdf]
